# Self-reported Antiretroviral Adherence: Association With Maternal Viral Load Suppression in Postpartum Women Living With HIV-1 From Promoting Maternal and Infant Survival Everywhere, a Randomized Controlled Trial in Sub-Saharan Africa and India

**DOI:** 10.1097/QAI.0000000000003102

**Published:** 2022-09-28

**Authors:** Neetal Nevrekar, Kevin Butler, David E. Shapiro, Patience Atuhaire, Taha E. Taha, Bonus Makanani, Lameck Chinula, Maxensia Owor, Dhayendre Moodley, Tsungai Chipato, Katie McCarthy, Patricia M. Flynn, Judith Currier, Mary Glenn Fowler, Amita Gupta, Nishi Suryavanshi

**Affiliations:** a Byramjee Jeejeebhoy Government Medical College- Johns Hopkins University Clinical Research Site, Pune, India;; b Center for Biostatistics in AIDS Research, Harvard T.H. Chan School of Public Health, Boston, MA;; c Makerere University–John Hopkins University Research Collaboration (MUJHU CARE LTD) CRS, Kampala, Uganda;; d Department of Epidemiology, Johns Hopkins University, Bloomberg School of Public Health, Baltimore, MD;; e College of Medicine-Johns Hopkins Project, Blantyre, Malawi;; f UNC Project Malawi CRS, Lilongwe, Malawi;; g Centre for the AIDS Programme of Research in South Africa and Department of Obstetrics and Gynaecology, School of Clinical Medicine, University of KwaZulu Natal, Durban, South Africa;; h Nelson R. Mandela School of Medicine, Durban, South Africa;; i Department of Obstetrics and Gynaecology, University of Zimbabwe, Harare, Zimbabwe;; j FHI 360, Durham, NC;; k Department of Infectious Diseases, St. Jude Children's Research Hospital, Memphis, TN;; l UCLA Centre for Clinical AIDS Research and Education, Los Angeles, CA;; m Department of Pathology, Johns Hopkins University, School of Medicine, Baltimore, MD; and; n Johns Hopkins University, School of Medicine, Baltimore, MD.

**Keywords:** mART, women with HIV, ART adherence, postpartum, viral load

## Abstract

Optimal adherence to antiretroviral therapy (ART) is crucial to promoting maternal–infant health.

**Setting:**

Fourteen sites in 7 countries within sub-Saharan Africa and India.

**Methods:**

The multicomponent, open-label strategy PROMISE trial enrolled breastfeeding mother–infant pairs not meeting in-country criteria for maternal ART (mART) initiation in the postpartum component within 5 days of delivery. Randomization was to mART versus infant NVP (iNVP) prophylaxis. Infants in the mART arm also received 6 weeks of iNVP. Self-reported adherence was assessed in a secondary analysis. Time-to-event analyses were performed to explore the association between adherence and maternal viral load (mVL) in the mART arm.

**Results:**

Two thousand four hundred thirty-one mother–infant pairs were enrolled between 2011 and 2014; the baseline maternal median CD4 was 686 (IQR 553–869), and the median mVL was 322 copies/mL (IQR 40–1422). Self-reported adherence was lower in the mART arm compared with the iNVP arm (no missed doses within 4 weeks of all study visits: 66% vs 83%; within 2 weeks: 71% vs 85%; *P* < 0.0001). The iNVP adherence at week 6 was high in both arms: 97% in mART arm; 95% in iNVP arm. Time-to-event analyses showed that adherence to mART was associated with time to first mVL ≥400 copies/mL (*P* < 0.0001). Missing 1 full day of doses over 3 days was associated with a 66% risk of mVL ≥1000 copies/mL (HR: 1.66; 95% CI: 1.37, 1.99).

**Conclusions:**

Postpartum women were less adherent to their own ART than mothers providing their infant's nevirapine prophylaxis. The self-reported missed mART doses were associated with high mVL. Strategies to optimize postpartum mART adherence are urgently needed.

**Clinical Trial Number:**

ClinicalTrials.gov: NCT01061151; closed to follow-up.

## INTRODUCTION

Globally, almost 50% of the 38 million adults living with human immunodeficiency virus (HIV) are women.^1^ The introduction and implementation of universal, lifelong ART for all pregnant and breastfeeding women through Option B+ since 2013 has substantially increased the number of HIV-positive women initiating ART.^2^ This ART strategy along with extended infant nevirapine prophylaxis has resulted in a marked decrease in mother-to-child transmission (MTCT).^3–5^ Despite these advances, adherence to ART among women with HIV remains a great concern in high-income and low-income countries. Pregnancy and postpartum bring about changes in a woman's life that may affect adherence to ART.^6^ Evidence suggests that achieving optimal ART adherence during pregnancy and postpartum can be challenging.^7–10^ The desire to prevent the transmission of HIV to the unborn child during pregnancy motivates women to adhere to ART.^11^ However, after delivery, caring for a newborn may be overwhelming, leaving little time for self-care in women. In addition, in the postpartum period, women may no longer be motivated by the need to prevent HIV transmission to her infant and that can affect their adherence to maternal ART (mART) and to remain in HIV care.^7,12,13^ A systematic review and meta-analysis of 51 studies from 4 countries found that the maternal ART adherence levels during postpartum was only 53% [95% confidence interval (CI) 32.8% to 72.7%; *P* = 0.005].^5,14,15^ In a large, observational cohort study of individuals enrolled in routine HIV care in Uganda and South Africa, the ART adherence in women with early-stage HIV infection was as high or higher than in those initiating ART with late-stage HIV infection. In Uganda, adherence was generally high and did not differ by the stage of ART initiation or pregnancy. In South Africa, ART adherence was higher in nonpregnant versus pregnant women.^16^

Measuring adherence is difficult because it often relies on the self-reported information by a patient. Self-reporting is fraught with recall and social desirability biases causing concerns about the validity of adherence measures.^17–19^ However, several studies substantiate these measures as appropriate for estimating adherence to ART in developing settings.^20,21^ The multicountry PEPFAR PROMISE Ongoing Treatment Evaluation (PROMOTE) cross-sectional study indicated that the self-reported missed ART doses (adjusted prevalence risk ratio 1.63, 95% CI: 1.24 to 2.13, *P* < 0.001) was associated with an increased risk of unsuppressed viremia.^21^ Although there is no standardized value, an ART adherence of 95% is critical for maintaining virologic suppression and optimizing health outcomes.^22,23^ Poor adherence in pregnant and breastfeeding women can increase the risk of virologic failure, maternal HIV disease progression, mother-to-child transmission (MTCT), and emergence of antiretroviral drug resistance.^24^ Detecting suboptimal adherence to ART is important because adherence-improving interventions may improve viral response, maternal health, and survival. We describe a longitudinal analysis of self-reported adherence to mART among mothers with HIV and to infant NVP (iNVP) among their infants exposed to HIV. Furthermore, we assess the association of adherence with maternal viral load suppression with the goal to inform the HIV treatment programs, which can devise effective strategies to optimize postpartum maternal ART adherence.

## METHODS

### Study Design and Settings

The International Maternal Pediatric Adolescent AIDS Clinical Trials (IMPAACT) Network's Promoting Maternal and Infant Survival Everywhere (PROMISE) 1077BF study was an open-label, multicomponent randomized controlled trial to determine the optimal mART and infant prophylaxis strategies to prevent MTCT and preserve maternal health and infant survival. Enrollments into this trial were conducted between June 2011 and October 2014 at 14 research sites in 7 countries (India, Malawi, South Africa, Tanzania, Uganda, Zambia, and Zimbabwe); the follow-up continued through September 2016. The postpartum component of PROMISE was designed to compare the efficacy and safety of triple mART versus daily iNVP prophylaxis for the prevention of MTCT through breastfeeding (BF), among women with CD4 cell count > 350 cells/mm^3^ (or the country-specific threshold for ART initiation, if that threshold was higher); a secondary objective was to compare adherence to mART and iNVP. Women who intended to breastfeed their infants were recruited in this component from 2 sources: women completing the PROMISE antepartum component and women first identified in early or active labor or within 5 days after delivery (“late presenters”) [Fowler et al., NEJM, 2016].

### Randomization

Eligible mother–infant pairs who consented for the postpartum component were randomized to 1 of the 2 arms: mART or iNVP at week 1 (day 6–14 postdelivery) continued until 18 months postdelivery or breastfeeding cessation, whichever occurred first unless stopped for toxicity, other medical reasons, or confirmed infant HIV infection. All infants received nevirapine during the first 6 weeks of life irrespective of the arms to which they were randomized. The infants in the mART arm received daily NVP only up to age 6 weeks as recommended by the World Health Organization (WHO) guidelines whereas the infants in the iNVP arm continued to receive NVP beyond 6 weeks of life through breastfeeding cessation or through 18 months postpartum, whichever came first unless it had to be stopped for infant HIV infection, toxicity, or other medical reasons. The preferred study–supplied mART regimen was twice daily protease inhibitors (PI) lopinavir/ritonavir (LPV/RTV) plus fixed-dose combination emtricitabine/tenofovir disoproxil fumarate (FTC/TDF) as the dual NRTI backbone. Combined ART regimens not provided by this study were allowed if they included at least 3 or more drugs from 2 or more antiretroviral classes.

### Procedures and Measures

Maternal postpartum visits occurred at weeks 1, 6, and 14 after delivery and then every 12 weeks through week 74. Infant visits occurred at postpartum weeks 1, 6, 10, 14, 18, 22, and 26, then every 12 weeks until week 98, with a final visit at week 104. Self-reported adherence was collected for all the mother–infant pairs at weeks 1, 6, 14, 26, 50, and 74 using a validated adherence questionnaire.^25,26^ The process of data collection by administering the adherence questionnaire was standardized across study sites. At each study visit, women in the mART arm were asked how many doses of ART they had missed in the last 3 days; they were also asked “When was the last time you missed any of your medications?” with the following responses offered: within the past week, 1–2 weeks ago, 2–4 weeks ago, 1–2 months ago, more than 2 months ago, and never missed medications. For women in the iNVP arm, the question asked was, “When was the last time you/your baby/your child missed a dose of any of the medications?” with the following responses offered: never, during the previous 2 weeks, during the last month, over a month ago, and do not remember. HIV-1 RNA was collected for all the women at weeks 1, 6, 14, 26, 50, and 74 postpartum and used for virologic monitoring. Virologic failure (VF) was defined as confirmed RNA level > 1000 copies/mL at or after 24 weeks on the ART regimen. Women with an elevated viral load (VL) were asked to return within 4 weeks for confirmatory testing. Women with confirmed VF were offered intensive adherence counselling and followed monthly with repeat VL assessment. All the mother–infant pairs in the PROMISE postpartum component in both the arms were included in the analysis. We evaluated the self-reported adherence to antiretroviral medications in the mART and iNVP arms and the association of adherence to mART with maternal viral load suppression. Adherence to maternal provision of iNVP through 6 weeks of life in the mART arm was also assessed.

### Statistical Analysis

This analysis was conducted to examine the association of self-reported adherence with maternal viral load suppression. The primary exposures of interest for this analysis were (1) self-reported adherence to mART and (2) self-reported adherence to iNVP for infants in both arms. The outcome of interest was lack of maternal virologic suppression defined as maternal viral load (mVL) of > 400 or > 1000 copies/mL at least 6 weeks after randomization.

The adherence responses were analyzed using dichotomous and continuous measures of adherence. A dichotomous measure of adherence was defined as missing any of the ART medication within a 4-, 2-, and 1-week period of each visit. A continuous measure of adherence at each visit was the proportion of missed doses within 3 days before study visit, defined as the total number of doses missed/total number of doses expected within a 3-day period at each visit. The dichotomous measures of adherence were combined across visits to get an overall measure of adherence. A χ^2^ test was performed to compare this overall adherence measure between postpartum treatment arms. The continuous adherence measure was combined across all drugs that the participant was taking at the time of the visit and was presented on a scale of 0–3, where each unit represented a full day of missed doses. We considered a response of “do not remember” to be a missing value in the analysis. Time-to-event analyses were performed to explore the association between maternal adherence and mVL. Using the dichotomous and continuous adherence measures as time-dependent predictors, proportional hazards regression analyses were performed, and hazard ratios (HR) and 95% confidence intervals were generated. The models analyzed the adherence measures as predictors of time to first maternal RNA ≥400 copies/mL and time to first maternal RNA ≥1000 copies/mL at least 6 weeks after randomization in the mART arm. The mVL outcomes from study visit week 6 through week 74 were summarized using the Kaplan–Meier survival curves. *P* values of < 0.05 were considered statistically significant. Analyses were conducted using SAS version 9.4 software (SAS Institute Inc; Cary, NC).

## RESULTS

### Characteristics of Study Population

The PROMISE study enrolled 2431 postpartum, breastfeeding mother–infant pairs in the postpartum component (Table 1). There were 1220 pairs randomized to the mART arm and 1211 to the iNVP arm from sites in Malawi (32%), South Africa (23%), Zimbabwe (22%), Uganda (16%), Zambia (2%), Tanzania (2%), and India (3%). Nearly all (1207, 98.9%) of the mothers in the mART arm started the preferred study–supplied mART regimen, and nearly all (1204, 98.9%) of the infants in the iNVP arm started on daily NVP. The median age at entry into PROMISE trial was 26 years [interquartile range (IQR) 23–30], 97% were in WHO clinical stage I, median CD4^+^ T-cell count was 686 cells/mm^3^ (IQR 553–869), and median mVL was 322 copies/mL (IQR 40–1422). There were 23% mothers in the mART arm and 24% in the iNVP arm with mVL< 400 copies/mL at the time of entry to the postpartum component. These baseline characteristics have been published in the primary PROMISE postpartum component manuscript (https://www.ncbi.nlm.nih.gov/pmc/articles/PMC5825265/).

**TABLE 1.** Baseline Maternal Characteristics

Maternal Characteristics	mART Arm (N = 1220)	iNVP Arm (N = 1211)	Total (N = 2431)
Age (yrs)			
Median (IQR)	26 (23–30)	26 (23–30)	26 (23–30)
Race			
Black African	1178 (97%)	1168 (96%)	2346 (97%)
Asian (Indian)	41 (3%)	42 (3%)	83 (3%)
Colored	1 (0%)	1 (0%)	2 (0%)
WHO clinical Stage I	1174 (96%)	1182 (98%)	2356 (97%)
Screening CD4 count (cells/mm^3^)			
Median (IQR)	682.5 (555–870)	691 (550–868)	686 (553–869)
HIV-1 viral load (copies/mL) [at delivery/week 1 postpartum]			
Median (IQR)	220 (40–1029)	400 (40–1960)	322 (40–1422)
<400	276 (23%)	296 (24%)	572 (24%)
On study ART regimen			
LPV/r based	98%	N/A	—
NNRTI	1%		

### Self-reported Adherence in mART

The self-reported adherence of the mothers to mART was between 82% and 86% in the mART arm for each postbaseline study visit week. It was lower compared with adherence to maternal provision of iNVP in the iNVP arm and was reported by mothers to be between 93% and 97% at all weeks (Table 2).

**TABLE 2.** Summary of Adherence Over Study Visits by Adherence Category

Study Visit Week	Missed Dose Within 4 wk	mART Arm (N = 1220)	iNVP Arm (N = 1211)	Total
Week 6 (N = 2354)	Never missed a dose	1003 (86%)	1104 (93%)	2107 (90%)
	Missed dose over 1 mo ago	12 (1%)	0 (0%)	12 (1%)
	Missed dose 2–4 wk ago	17 (1%)	4 (0%)	21 (1%)
	Missed dose within the last 2 wk	140 (12%)	74 (6%)	214 (9%)
	Total	1172	1182	2384
Week 14 (N = 2263)	Never missed a dose	956 (85%)	1081 (95%)	2037 (90%)
	Missed dose over 1 mo ago	20 (2%)	0 (0%)	20 (1%)
	Missed dose 2–4 wk ago	35 (3%)	9 (1%)	44 (2%)
	Missed dose within the last 2 wk	112 (10%)	50 (4%)	162 (7%)
	Total	1123	1140	2263
Week 26 (N = 2161)	Never missed a dose	888 (83%)	1035 (95%)	1923 (89%)
	Missed dose over 1 mo ago	48 (4%)	1 (0%)	49 (2%)
	Missed dose 2–4 wk ago	31 (3%)	8 (1%)	39 (2%)
	Missed dose within the last 2 wk	103 (10%)	47 (4%)	150 (7%)
	Total	1070	1091	2161
Week 50 (N = 1738)	Never missed a dose	716 (84%)	841 (95%)	1557 (90%)
	Missed dose over 1 mo ago	37 (4%)	9 (1%)	46 (3%)
	Missed dose 2–4 wk ago	34 (4%)	7 (1%)	41 (2%)
	Missed dose within the last 2 wk	64 (8%)	30 (3%)	94 (5%)
	Total	851	887	1738
Week 74 (N = 766)	Never missed a dose	311 (82%)	377 (97%)	688 (90%)
	Missed dose over 1 mo ago	15 (4%)	2 (1%)	17 (2%)
	Missed dose 2–4 wk ago	17 (5%)	1 (0%)	18 (2%)
	Missed dose within the last 2 wk	34 (9%)	9 (2%)	43 (6%)
	Total	377	389	766

Table 3 summarizes the comparison of dichotomous adherence measures between the mART arm and the iNVP arm combined over all postenrollment visit weeks. Self-reported adherence to mART with the dichotomous measure was lower than adherence to iNVP within 4 and 2 weeks before study visits. The proportion of study participants reporting no missed doses within 4 weeks of all visits was 66% versus 83% and within 2 weeks was 71% versus 85% comparing mART versus iNVP for both periods (*P* < 0.0001).

**TABLE 3.** Self-Reported Adherence With Dichotomous Measures Combined Over all Study Visits Using χ^2^ Test

Adherence Measure	mART Arm (N = 1220)	iNVP Arm (N = 1211)	*P*
Did not miss any dose within the last 4 wk at any study visit	776 (66%)	987 (83%)	<0.0001
Missed a dose within the last 4 wk during any study visit	403 (34%)	198 (17%)	
Total	1179	1185	
Did not miss any dose within the last 2 wk at any study visit	835 (71%)	1010 (85%)	<0.0001
Missed a dose within the last 2 wk during any study visit	343 (29%)	175 (15%)	
Total	1178	1185	

The mean proportions of doses missed in the last 3 days for each study visit measured for mothers in the mART arm and for infants in the iNVP arm as a continuous adherence measure were low in both arms (3%–4%).

### Self-reported Adherence in the iNVP Arm

Self-reported adherence to maternal provision of infant NVP at week 6 was high in both the arms: 97% in the mART arm and 95% in the iNVP arm (Table 4). It was not significantly different across arms (*P* = 0.066 for within 4 weeks and *P* = 0.074 for within 2 weeks). The self-reported adherence to infant NVP by dichotomous measure at the week 6 visit was higher than the mothers' adherence to mART: 97% versus 66% within 4 weeks and 97% vs 71% within 2 weeks (Table 4). The self-reported adherence to infant NVP with the continuous measure was high in both the arms and similar to those with dichotomous measure.

**TABLE 4.** Comparison of Adherence for Infants on NVP at Week 6

Study Visit Week	Adherence Measures	mART Arm (N = 1220)	iNVP Arm (N = 1211)	*P*
Week 6 (N = 2277)	Did not miss any dose within the last 4 wk	1072 (97%)	1109 (95%)	0.066
	Missed a dose within the last 4 wk	38 (3%)	58 (5%)	
	Total	1110	1167	
Week 6 (N = 2277)	Did not miss any dose within the last 2 wk	1074 (97%)	1112 (95%)	0.074
	Missed a dose within the last 2 wk	36 (3%)	55 (5%)	
	Total	1110	1167	

### Association of Time-Dependent Adherence with mVL

The analyses of the association of time-dependent mART adherence measured as a dichotomous variable and as a continuous variable with time to first mVL measurement ≥400 copies/mL and ≥1000 copies/mL in the mART arm are summarized in Table 5. The analysis of the dichotomous measure shows that there was no significant association between maternal adherence to mART and time to first occurrence of mVL ≥ 400 copies/mL or ≥1000 copies/mL. However, the continuous measure analyses showed a significant association between maternal adherence to mART and time to first mVL ≥400 copies/mL or ≥1000 copies/mL. Missing 1 full day of mART doses over the past 3 days before a study visit was associated with a 58% higher risk of having a mVL ≥400 copies/mL (hazard ratio (HR): 1.58; 95% CI: 1.33 to 1.87) and 66% risk of mVL ≥1000 copies/mL (HR: 1.66; 95% CI: 1.37 to 1.99) (Table 5).

**TABLE 5.** Time-To-Event Analysis With Adherence as a Time-Varying Predictor of mVL in the mART Arm

	Hazard Ratio (95% CI)	Standard Error	*P*
Outcome I mVL ≥ 400 copies/mL			
Missed dose within 4 wk of visit	0.97 (0.75 to 1.25)	0.131	0.80
Missed dose within 2 wk of visit	1.04 (0.78 to 1.39)	0.149	0.80
Missed dose within 1 wk of visit	1.08 (0.74 to 1.58)	0.193	0.69
*Total doses missed/total doses expected over past 3 d	1.58 (1.33 to 1.87)	0.088	<0.0001
Outcome II mVL ≥ 1000 copies/mL			
Missed dose within 4 wk of visit	1.11(0.83 to 1.48)	0.147	0.47
Missed dose within 2 wk of visit	1.15 (0.83 to 1.60)	0.169	0.40
Missed dose within 1 wk of visit	1.21 (0.80 to 1.86)	0.216	0.37
*Total doses missed/total doses expected over past 3 d	1.66 (1.37 to 1.99)	0.095	<0.0001

* A 1 unit change in adherence interprets as missing 1 full day of drug within the last 3 day.

## DISCUSSION

This longitudinal analysis examined the association between self-reported adherence and maternal viral load suppression in sub-Saharan Africa and India. Our analysis showed that more than three-fourths of women in the mART arm reported high adherence to their antiretrovirals at all postpartum study weeks. Although an adherence to ART of 95% is desirable for viral suppression,^8,27^ the 86% adherence level in this study is higher than that reported among postpartum mothers in a study in western Uganda^28^ and similar to the Breastfeeding, Antiretrovirals, and Nutrition study, conducted in Lilongwe, Malawi.^14^ Of concern, however, was the lower self-reported maternal adherence to mART than self-reported adherence to maternal provision of iNVP by dichotomous measures at all study visits. This is potentially due to the relative complexity of the 3 times a day maternal ART regimen compared with the once daily iNVP prophylaxis and/or to mothers prioritizing infant's HIV prophylaxis over their own HIV treatment to prevent MTCT of infection. Previous studies have suggested that the disparity in adherence to mART and iNVP may be due to lack of self-motivation in mothers especially after the infant's HIV test result was negative along with the maternal belief that HIV care for her own health is not as important as the care of the newborn.^5,29,30^ Our finding concurs with a longitudinal study by Larsen et al from South Africa which observed higher infant adherence to prophylaxis than maternal adherence throughout the 18 months postpartum besides the suboptimal adherence among both mothers and infants.^31^ By contrast, the Breastfeeding, Antiretrovirals, and Nutrition study in Malawi did not find any difference between the maternal adherence to her own ART and provision of iNVP.^14^

The self-reported adherence in the 3 days preceding the study visits by the continuous adherence measure was high in both arms, although again, the mothers in the mART arm reported higher adherence to their infant's NVP than their own mART. This was comparable with a cross-sectional study conducted in South Africa wherein 86% of postnatal women reported complete adherence to the zidovudine medication schedule in the 4 days preceding the visit.^32^

With half of all MTCT occurring during breastfeeding, making this a crucial time for viral suppression, ongoing VL monitoring throughout this period is recommended to help minimize postpartum transmission risks.^33^ However, not all settings are able to implement such frequent viral load testing.^34,35^ Assessing ART adherence may help to take the necessary clinical measures and institute intensive adherence interventions between viral load tests with the goal of achieving viral suppression.

The time-to-event analyses were conducted to see whether self-reported maternal adherence to mART can predict time to first mVL ≥ 400 or ≥1000 copies/mL. We found that missing a dose of mART within the last 4 or 2 weeks because the dichotomized, self-reported adherence variable was not associated with time to first mVL occurrence over 400 or 1000 copies/mL at least 6 weeks after randomization. However, notably, with the continuous adherence measure, we observed an association between self-reported missed mART doses over the last 3 days preceding the study visit with time to first occurrence of mVL ≥ 400 or ≥1000 copies/mL. Our findings suggest that this adherence measure can be used as an interim screening tool in settings where resources limit the frequent VL monitoring. This will allow for rapid assessment of decreased adherence risk and help in providing prompt adherence counselling and interventions.

Studies conducted in South Africa and in Cape Town, evaluating self-reported adherence questionnaire and scale in HIV-infected adults, found no correlation between adherence and VL.^36,37^ Both these studies used recall periods of 7 days as against the 3-day period used in our analysis.

The self-reported adherence and viral load measures were taken as part of PROMISE research study visits, independent of routine HIV care, with a large sample size using the same standardized process and questionnaire across all study sites. This accounts for the confidence in our data and may have reduced socially desirable response bias.

### Limitations

The PROMISE study did not assess some of the important barriers to ART adherence in the postpartum period such as HIV disclosure, intimate partner violence, and depression, and therefore, these factors could not be included in the analyses. These important factors are known to affect adherence.^38–40^ The most common ART regimen used in our study was lopinavir/ritonavir, which at the time was a first-line regimen in the United States, but which is no longer used as standard of care in pregnancy or postpartum. Newer integrase inhibitor regimens as the first-line ART and for prevention of mother-to-child transmission are now available. Owing to their higher efficacy, good tolerability, simpler dosing regimen, and relatively higher barrier to HIV drug resistance, these regimens are soon becoming standard of care in many settings.^41,42^ With increased access to integrase-based regimens, it is possible that the postpartum women will have further improved adherence and be at lower risk of virologic failure. Our measurement of ART adherence is based on self-report of missed ART doses which may be prone to recall and social desirability biases. Self-reported adherence is known to overestimate adherence compared with objective measures.^43–45^

## CONCLUSIONS

In conclusion, our results show that the self-reported missed mART doses were associated with increased risk of unsuppressed mVL. Our findings also highlight the need for individual counselling and education regarding the importance of adhering to ART for the mother's own health after delivery. Suboptimal mART adherence warrants timely attention and intervention for successful treatment outcomes. As the ART programs across the globe seek to expand ART access, they should facilitate optimal adherence to ART by addressing potential barriers that are unique to the postpartum period. Women should be closely monitored and counselled about the importance of ART in preventing disease progression. We suggest that effective interventions should be designed to improve adherence among postpartum women for long-term benefits of ART use and sustained viral suppression.

Future research should focus on integrating behavioral interventions and individual counselling to maximizing ART adherence and for better clinical outcomes.
